# The Effect of Number and Position of P=O/P=S Bridging Units on Cavitand Selectivity toward Methyl Ammonium Salts

**DOI:** 10.3390/molecules20034460

**Published:** 2015-03-10

**Authors:** Daniela Menozzi, Roberta Pinalli, Chiara Massera, Francesca Maffei, Enrico Dalcanale

**Affiliations:** Dipartimento di Chimica, Università di Parma and INSTM UdR Parma, Parco Area delle Scienze 17/A, 43124 Parma, Italy; E-Mails: Daniela.Menozzi@altana.com (D.M.); roberta.pinalli@unipr.it (R.P.); chiara.massera@unipr.it (C.M.); framaffy@yahoo.com (F.M.)

**Keywords:** phosphonate cavitands, thiophosphonate cavitands, mixed-bridged cavitands, molecular recognition, *N,N*-methyl alkyl ammonium salts, ITC

## Abstract

The present work reports the synthesis and complexation properties of five mixed bridge P=O/P=S cavitands toward *N,N*-methyl butyl ammonium chloride (**1**) as prototype guest. The influence of number and position of P=O and P=S groups on the affinity of phosphonate cavitands toward **1** is assessed via ITC titrations in DCE as solvent. Comparison of the resulting Kass values, the enthalpic and entropic contributions to the overall binding with those of the parent tetraphosphonate **Tiiii** and tetrathiophosphonate **TSiiii** cavitands allows one to single out the simultaneous dual H-bond between the cavitand and the salt as the major player in complexation.

## 1. Introduction

Tetraphosphonate cavitands represent an interesting class of synthetic receptors featuring peculiar molecular recognition properties toward methyl ammonium guests [1,2]. The origin of this selectivity has been identified in the synergistic presence of three different interactions, namely (i) N^+^•••O=P cation-dipole interactions; (ii) CH_3_-π interactions of the acidic ^+^N-CH_3_ group with the π basic cavity [3]; (iii) two simultaneous hydrogen bonds between two adjacent P=O bridges and the two nitrogen protons. The resulting unique selectivity toward methyl ammonium salts has been exploited in devices for the detection of sarcosine in urine [4] and illicit drugs in water [5].

One key issue which remains to be addressed is which is the influence of number and relative position of the P=O bridges on the receptor selectivity. Complete substitution of the P=O units with either P=S or methylene bridges has been shown to completely switch off complexation toward this class of guests [6,7].

Previously, mixed-bridged phosphonate cavitands, featuring both P=O and P=S bridges, have been investigated in the context of alcohol sensing with Quartz Crystal Microbalance (QCM) transducers [8]. The progressive substitution of the P=O units with the bulkier, H-bond silent P=S ones led to a change of the sensor selectivity pattern with concomitant reduction of the sensitivity.

**Figure 1:**
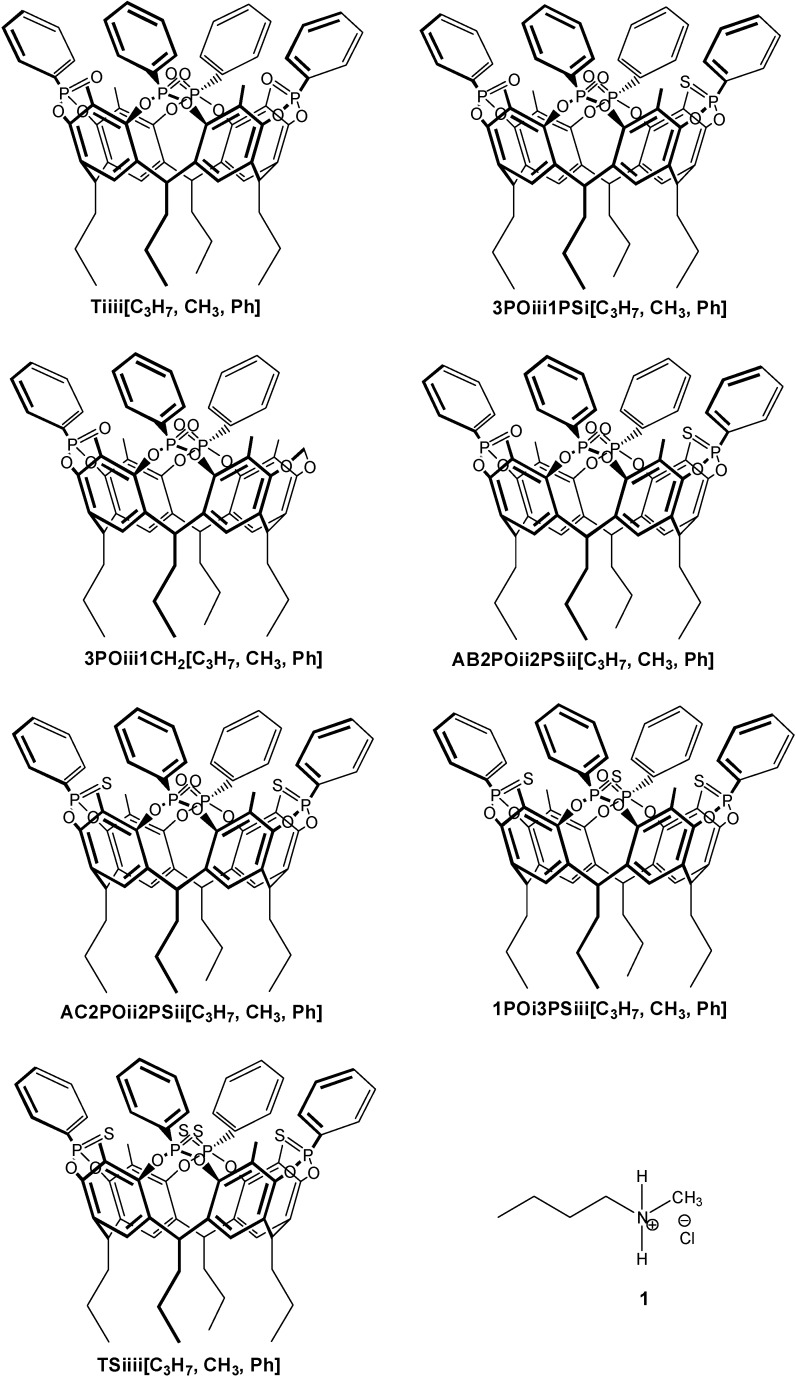
Molecular structures of cavitands and of the guest used in the present work.

In the present work we investigate the influence of number and position of P=O and P=S groups on the affinity of phosphonate cavitands toward methyl ammonium salts as target guests. The P=S group is known to be a weak H-bond acceptor compared to the P=O counterpart [9]. To this purpose all four mixed-bridged cavitands with inward facing P=O/P=S groups were synthetized and their complexation properties tested toward *N,N*-butyl methyl ammonium chloride as prototype of the preferred class of guests (Figure 1). Their binding properties are compared to those of the parent tetraphosphonate (**Tiiii**) and tetrathiophosphonate (**TSiiii)** cavitands to obtain a meaningful trend. The whole set of measurements is conducted *via* ITC in order to weight the enthalpic and entropic contributions to the overall binding. The binding ability of **Tiiii** toward the butyl ammonium chloride series has been qualitatively tested using ^31^P-NMR (see Figure S1, Supplementary Material).

## 2. Results and Discussion

### 2.1. Synthesis

**Scheme 1:**
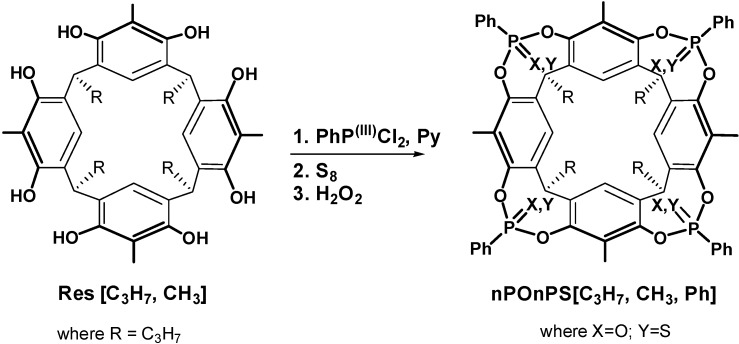
Synthesis of mixed PO/PS cavitand hosts.

**Scheme 2:**
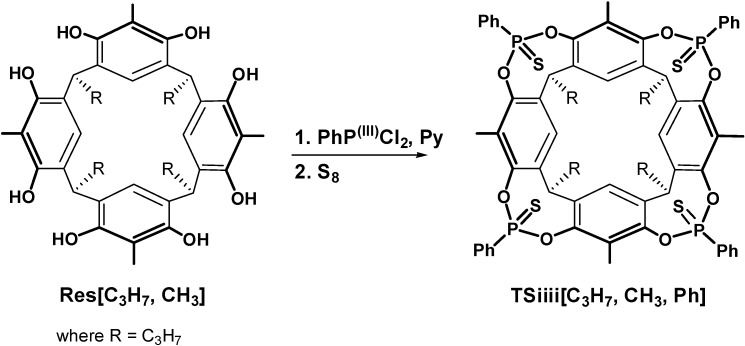
Synthesis of **TSiiii[C_3_H_7_**, **CH_3_**, **Ph]**.

**Scheme 3:**
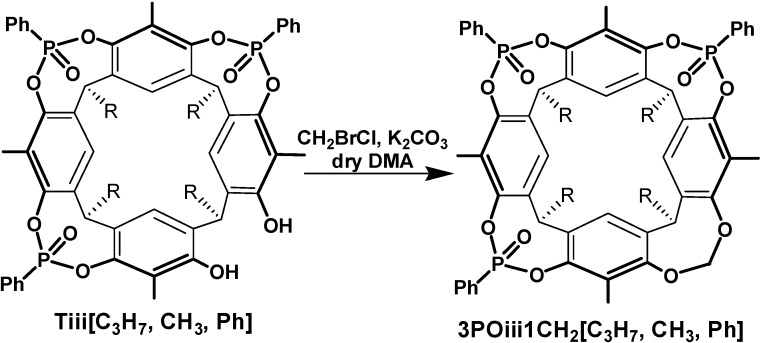
Synthesis of **3POiii1CH_2_[C_3_H_7_**, **CH_3_**, **Ph]**.

All cavitands prepared in this work have propyl feet to impart solubility in organic solvents. The preparation of cavitand **Tiiii[C_3_H_7_**, **CH_3_**, **Ph]** (from now on referred to as **Tiiii**) has been already reported [1]. Cavitands **3POiii1PSi[C_3_H_7_**, **CH_3_**, **Ph]**, **AB2POii2PSii[C_3_H_7_**, **CH_3_**, **Ph]**, **AC2POii2PSii[C_3_H_7_**, **CH_3_**, **Ph]**, **1POi3PSiii[C_3_H_7_**, **CH_3_**, **Ph]** are prepared via a one-step procedure starting from resorcinarene **Res[C_3_H_7_**, **CH_3_]** (Scheme 1). The resorcinarene is bridged with dichlorophenylphosphine and then oxidized *in situ* with S_8_ and hydrogen peroxide added in two steps in different stoichiometric ratios in order to favor the formation of one of the desired products. **AB2POii2PSii** and **AC2POii2PSii** cavitands are obtained in the same bridging reaction and then isolated via column chromatography. **TSiiii[C_3_H_7_**, **CH_3_**, **Ph]** (from now on referred to as **TSiiii**) is obtained via bridging reaction of **Res[C_3_H_7_**, **CH_3_]** with dichlorophenylphosphine and then oxidized *in situ* with S_8_ (Scheme 2). Host **3POiii1CH_2_[C_3_H_7_**, **CH_3_**, **Ph]** is synthesized via a one-step procedure via a bridging reaction with CH_2_BrCl of the residual phenolic OHs of the tri-phosphonate resorcinarene **Tiii[C_3_H_7_**, **CH_3_**, **Ph]** (Scheme 3).

### 2.2. Crystal Structure of the **1POi3PSiii[C_2_H_5_**, **CH_3_**, **Ph]** Cavitand

**Figure 2:**
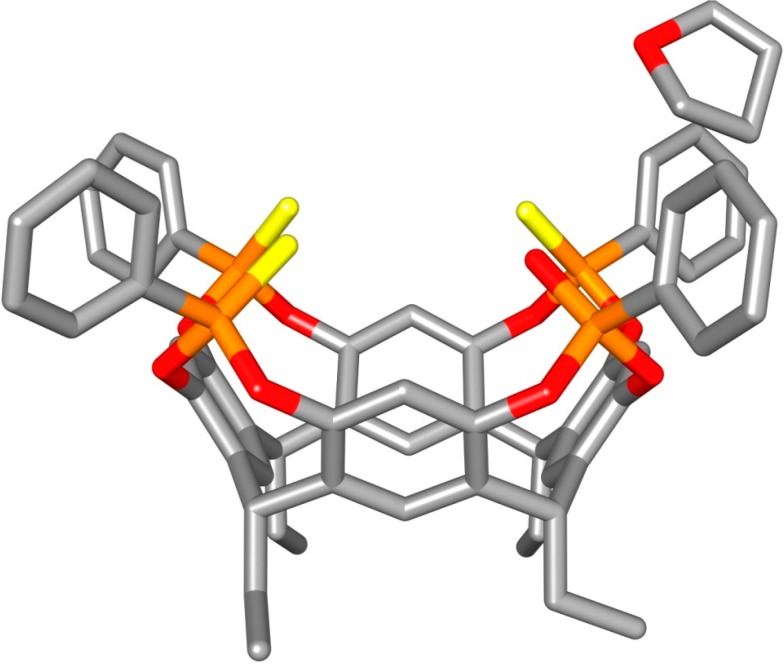
Molecular structure of **1POi3PSiii[C_2_H_5_**, **H**, **Ph]·3C_4_H_8_O**. Color code: P, orange; S, yellow; O, red; C, grey. Hydrogen atoms have been omitted for clarity. The two THF lattice molecules treated with SQUEEZE are not shown.

The crystal structures of the methanol complexes of **AB2POii2PSii** and **AC2POii2PSii** without alkyl feet have been already published [8]. Here we report the molecular structure of one of the two mixed-bridged cavitands not structurally characterized so far, namely the **1POi3PSiii**. Crystals of compound **1POi3PSiii[C_2_H_5_**, **H**, **Ph]·3C_4_H_8_O** were obtained from slow evaporation of a THF solution. The shorter ethyl feet at the lower rim allowed the formation of X-ray quality crystals. X-ray diffraction analysis on the single crystals confirmed the structure of a mixed 3P=S/1P=O cavitand (Figure 2), with P=S and P=O distances of 1.902(2), 1.891(1), 1.892(1) and 1.722(3) Å, respectively. The bulkiness of the three sulphur atoms prevents the inclusion of THF in the cavity. The solvent stabilizes the crystal structure occupying the voids in the lattice.

### 2.3. ITC Measurements

A comprehensive set of ITC complexation measurements was performed on all cavitands towards *N,N*-methyl butyl ammonium chloride (**1**) using dichloroethane (DCE) as solvent (Table 1). Thermodynamic parameters of the host-guest interactions (the equilibrium constant Kass, and the changes in enthalpy, entropy, and free energy ∆H, ∆S, and ∆G) were extrapolated from the binding curves. The single-site (monovalent) model to fit the binding curve was adopted, supported by the crystal structures of several related complexes [3,4]. Several considerations can be made by comparing the ITC titrations of the seven cavitands with guest **1**.

The ∆H and T∆S results indicate that the complexation of **1** is both enthalpy and entropy driven for four out of five effective receptors, while for the last one (**3POiii1CH_2_**) it is entropy neutral. The unusual entropic gain can be coarsely interpreted in terms of an increase in solvent entropy associated with the desolvation (viz., solvent displacement) of both host and guest upon complexation. For **TSiiii** and **1POi3PSiii** the interaction with **1** is either absent or too low to be measured, respectively.

This clearly indicates that the replacement of three/four P=O with P=S suppresses complexation, highlighting the pivotal role of multiple H-bonding. At the opposite end of the spectrum there is the behavior of the three cavitands **Tiiii**, **3POiii1PSi** and **AB2POii2PSii**, which present comparable Kass values in the 10^5^ range, with **3POiii1PSi** at the higher end of the range. Replacement of a single P=O with a P=S (cfr **3POiii1PSi** with **Tiiii**) does not influence significantly the complexation, since all the interaction modes enumerated in the introduction are still present. Interestingly, there is an enthalpic-entropic compensation in moving from **1@Tiiii** to **1@3POiii1PSi** and to **1@AB2POii2PSii**, *i.e.*, in replacing one/two P=O with P=S. The entropic reduction trend can be explained recalling that there is a sizable entropic gain experienced by the guest upon H-bond interactions with multiple energetically equivalent P=O acceptor sites, as demonstrated in the gas phase [2] and in alcohol complexation [10]. The reverse enthalpic gain can be rationalized considering that the bulkier P=S units force the guest to be closer to the P=O groups, thus strengthening the H-bonding.

**Table 1:** Results of ITC titrations of guest 1 with hosts **Tiiii[C_3_H_7_**, **CH_3_**, **Ph]**, **3POiii1PSi[C_3_H_7_**, **CH_3_**, **Ph]**, **3POiii1CH_2_[C_3_H_7_**, **CH_3_**, **Ph]**, **AB2POii2PSii[C_3_H_7_**, **CH_3_**, **Ph]**, **AC2POii2PSii[C_3_H_7_**, **CH_3_**, **Ph]**, **1POi3PSiii[C_3_H_7_**, **CH_3_**, **Ph]** and **TSiiii[C_3_H_7_**, **CH_3_**, **Ph]** in DCE at 303 K.

Guest	Host		Solvent	Kass ± δKass (M^−1^)	ΔH ± δH (KJ·mol^−1^)	ΔG± δG (KJ·mol^−1^)	TΔS ± TδS (KJ·mol^−1^)
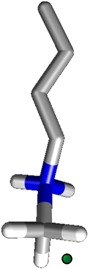 *N*-methylbutyl ammonium chloride **1**	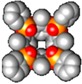	Tiiii[C_3_H_7_, CH_3_, Ph]	DCE	(2.04 ± 0.2) × 10^5^	−19.04 ± 0.2	−30.80 ± 1.8	11.76 ± 3.4
	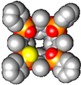	3POiii1PSi[C_3_H_7_, CH_3_, Ph]	DCE	(4.95 ± 0.4) × 10^5^	−24.2 ± 0.2	−33.03 ± 0.1	8.75 ± 0.1
	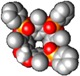	3POiii1CH_2_[C_3_H_7_, CH_3_, Ph]	DCE	(2.05 ± 0.1) × 10^4^	−24.86 ± 0.1	−25.01 ± 0.7	0.15 ± 1.2
	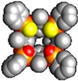	ABPOii2PSii[C_3_H_7_, CH_3_, Ph]	DCE	(1.63 ± 0.1) × 10^5^	−28.21 ± 0.2	−30.24 ± 0.6	2.02 ± 0.8
	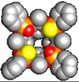	ACPOii2PSii[C_3_H_7_, CH_3_, Ph]	DCE	(2.78 ± 0.1) × 10^3^	−11.22 ± 0.1	−19.98 ± 1.8	8.76 ± 3.0
	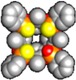	1POi3PSiii[C_3_H_7_, CH_3_, Ph]	DCE	Interaction too low to be measured			
	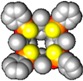	TSiii[C_3_H_7_, CH_3_, Ph]	DCE	Interaction not detectable			

The influence of P=O positioning over the cavity is evidenced by the comparison between the **AB2POii2PSii** and **AC2POii2PSii** isomers, having the two P=O units placed, respectively, vicinal and distal. The **AB** cavitand shows a comparable Kass with respect to **Tiiii**, mainly enthalpic in origin, demonstrating that two P=O units are sufficient for strong guest complexation when they interact simultaneously. In fact, as demonstrated in the gas phase [2], the H-bonding takes place with two adjacent P=O groups at a time. Instead, in the **AC** cavitand, the two P=O are too far apart to allow simultaneous H-bonds, leading to a significant drop in the association constant due to the partial loss of one H-bond. This loss is reflected in the drop of the enthalpic contribution of 17 KJ·mol^−1^.

The removal of a third P=O bridge in favor of a P=S (**1POi3PSiii**) leads to a collapse of the Kass below the limit of ITC sensitivity [11]. Therefore, the presence of a single H-bond assisted by CH_3_-π interactions is not sufficient for a decent complexation.

Replacement of a single P=S unit with a methylene bridge (cfr **3POiii1PSi** with **3POiii1CH_2_**) leads to one order of magnitude decrease in binding, ascribable entirely to a remarkable loss in the entropic contribution (8.6 KJ mol^−1^). This result indicates that the P=S bridge plays a significant role in reducing the cavity size, thus maximizing the desolvation of both the host and the guest upon complexation and reducing the guest mobility within the cavity. Instead, the comparable enthalpic values in the two complexes indicate that the P=S contribution to cation-dipole interactions is negligible.

## 3. Experimental Section

### 3.1. General Methods

All commercial reagents were ACS reagent grade and used as received. Pyridine was distilled using standard procedures. Flash column chromatography was carried out using Kieselgel C60 (Merck, Darmstadt, Germany) as the stationary phase. Analytical TLC was performed on precoated silica gel plates (0.25 mm thick, 60F254, Merck) and observed under UV light. ^1^H-NMR spectra were recorded on Bruker Avance 300 (300 MHz) and 400 (400 MHz) NMR spectrometers. All chemical shifts (d) were reported in parts per million (ppm) relative to proton resonances resulting from incomplete deuteration of NMR solvents. ^31^P-NMR spectra were recorded on a Bruker Avance 400 (162 MHz) NMR spectrometer, and all chemical shifts were reported to external 85% H_3_PO_3_ at 0 ppm. Electrospray ionization mass spectrometry (ESI-MS) experiments were performed on an API 100 SCIEX instrument with an electrospray interface.

ITC measurements were performed with a fully computer-operated MicroCal ITC-MCS instrument at 303K by adding 2–10 mL aliquots of the guest solution into the thermostated solution of the host compound present in about 10-15-fold lower concentration in the calorimetric cell (1.35 mL). To account for unspecific heats of dilution, each guest was also titrated into pure DCE (blank titration). In all cases, the signal from blank titrations was negligible with respect to the binding signal. Each experiment was replicated at least three times. Cavitands **Tiiii[C_3_H_7_**, **CH_3_**, **Ph]**, **Res[C_3_H_7_**, **CH_3_]** and guest **1** were prepared as described previously [1]. Tri-bridged resorcinarene **Tiii[C_3_H_7_**, **CH_3_**, **Ph]** was prepared following a published procedure [12].

### 3.2. Synthesis of Cavitand Hosts

#### 3.2.1. Cavitand **3POiii1PSi[C_3_H_7_**, **CH_3_**, **Ph]**

To a solution of resorcinarene **Res[C_3_H_7_**, **CH_3_]** (1 g, 1.40 mmol) in freshly distilled pyridine (30 mL), dichlorophenylphosphine (0.773 mL, 5.70 mmol) was added slowly under argon, and the reaction was kept under stirring at 70 °C. After 1 h S_8_ (45 mg, 0.18 mmol) was added and the reaction was stirred for another hour at 50 °C. After cooling to room temperature, the solution was treated with an excess of H_2_O_2_, added dropwise at 0 °C, and the reaction was stirred for 1 h at room temperature. Distilled water was then added to the reaction, favoring the formation of a white precipitate that was filtrated and washed with water. The crude was purified by column chromatography (SiO_2_, CH_2_Cl_2_:MeOH 95:5) to give the desired product with a 68% yield. ^1^H-NMR (CDCl_3_, 300 MHz): δ (ppm) 8.26–8.19 (m, 2H, PSArH*_o_*), 8.16–8.08 (m, 6H, POArH*_o_*), 7.69–7.53 (m, 12H, PSArH*_m_* + PSArH*_p_* + POArH*_m_* + POArH*_p_*), 7.26–7.24 (d, 4H, ArH*_down_*, *J* = 7.7 Hz), 4.84–4.78 (m, 4H, ArC*H*), 2.38–2.36 (m, 8H, C*H_2_*CH_2_CH_3_), 2.19–2.15 (s+s, 12H, ArC*H_3_*), 1.51–1.40 (m, 8H, CH_2_C*H_2_*CH_3_), 1.24–1.05 (m, 12H, CH_2_CH_2_C*H_3_*). ^31^P-NMR (CDCl_3_, 162 MHz): δ (ppm) 74.09 (s, 1P, P=S), 8.21 (s, 1P, P=O), 7.70 (s, 2P, P=O). ESI-MS: *m*/*z* 1239.0 [M+Na]^+^.

#### 3.2.2. Cavitand **AB2POii2PSii[C_3_H_7_**, **CH_3_**, **Ph]** and **AC2POii2PSii[C_3_H_7_**, **CH_3_**, **Ph]**

To a solution of resorcinarene **Res[C_3_H_7_**, **CH_3_]** (1 g, 1.40 mmol) in freshly distilled pyridine (30 mL), dichlorophenylphosphine (0.773 mL, 5.70 mmol) was added slowly under argon, and the reaction was kept under stirring at 70 °C. After 1 h S_8_ (112 mg, 0.44 mmol) was added and the reaction was stirred for another hour at 50 °C. After cooling to room temperature, the solution was treated with an excess of H_2_O_2_, added dropwise at 0 °C, and the reaction was stirred for 1 h at room temperature. H_2_O was then added to the reaction, favoring the formation of a white precipitate that was filtrated and washed with water. The crude was purified by column chromatography (SiO_2_, CH_2_Cl_2_:MeOH 95:5) to give the two desired isomers with a 20% yield for the **AB** and 25% for the **AC**. (**AB**) ^1^H-NMR (CDCl_3_, 300 MHz): δ (ppm) 8.23–8.08 (m, 8H, PSArH*_o_* + POArH*_o_*), 7.64–7.55 (m, 12H, PSArH*_m_* + PSArH*_p_* + POArH*_m_* + POArH*_p_*), 7.37 (bm, 4H, ArH*_down_*), 4.82–4.74 (m, 4H, ArC*H*), 2.38 (bm, 8H, C*H_2_*CH_2_CH_3_), 2.14–2.13 (m, 12H, ArC*H_3_*), 1.44 (bm, 8H, CH_2_C*H_2_*CH_3_), 1.10–1.03 (m, 12H, CH_2_CH_2_C*H_3_*). ^31^P-NMR (CDCl_3_, 162 MHz): δ (ppm) 75.05 (s, 2P, P=S), 7.61 (s, 2P, P=O). ESI-MS: *m*/*z* 1255.1 [M+Na]^+^. **(AC)**^1^H-NMR (CDCl_3_, 300 MHz): δ (ppm) 8.23–8.01 (m, 8H, PSArH*_o_* + POArH*_o_*), 7.67–7.54 (m, 12H, PSArH*_m_* + PSArH*_p_* + POArH*_m_* + POArH*_p_*), 7.23 (s, 4H, ArH*_down_*), 4.90–4.67 (bt+bt, 4H, ArC*H*), 2.41–2.24 (m, 8H, C*H_2_*CH_2_CH_3_), 2.14 (s, 12H, ArC*H_3_*), 1.50–1.35 (m, 8H, CH_2_C*H_2_*CH_3_), 1.24–1.01 (m, 12H, CH_2_CH_2_C*H_3_*). ^31^P-NMR (CDCl_3_, 162 MHz): δ (ppm) 74.62 (s, 2P, P=S), 7.23 (s, 2P, P=O). ESI-MS: *m*/*z* 1255.3 [M+Na]^+^.

#### 3.2.3. Cavitand **1POiii3PSi[C_3_H_7_**, **CH_3_**, **Ph]**

To a solution of resorcinarene **Res[C_3_H_7_**, **CH_3_]** (1 g, 1.40 mmol) in freshly distilled pyridine (30 mL), dichlorophenylphosphine (0.773 mL, 5.70 mmol) was added slowly under argon, and the reaction was kept under stirring at 70 °C. After 1 h S_8_ (209 mg, 0.82 mmol) was added and the reaction was stirred for another hour at 50 °C. After cooling to room temperature, the solution was treated with an excess of H_2_O_2_, added dropwise at 0 °C, and the reaction was stirred for 1 h at room temperature. H_2_O was then added to the reaction, favoring the formation of a white precipitate that was filtrated and washed with water. The crude was purified by column chromatography (SiO_2_, CH_2_Cl_2_:MeOH 9:1) to give the desired product with a 68% yield. ^1^H-NMR (CDCl_3_, 300 MHz): δ (ppm) 8.24–8.04 (m, 8H, PSArH*_o_* + POArH*_o_*), 7.67–7.50 (m, 12H, PSArH*_m_* + PSArH*_p_* + POArH*_m_* + POArH*_p_*), 7.26–7.24 (m, 4H, ArH*_down_*), 4.85–4.71 (m, 4H, ArC*H*), 2.38–2.30 (m, 8H, C*H_2_*CH_2_CH_3_), 2.14–2.12 (s+s, 12H, ArC*H_3_*), 1.54–1.41 (m, 8H, CH_2_C*H_2_*CH_3_), 1.17–0.96 (m, 12H, CH_2_CH_2_C*H_3_*). ^31^P-NMR (CDCl_3_, 162 MHz): δ (ppm) 75.02 (s, 1P, P=S), 74.34 (s, 2P, P=S), 7.18 (s, 1P, P=O). ESI-MS: *m*/*z* 1271.9 [M+Na]^+^.

#### 3.2.4. Cavitand **TSiiii[C_3_H_7_**, **CH_3_**, **Ph]**

To a solution of resorcinarene **Res[C_3_H_7_**, **CH_3_]** (1 g, 1.40 mmol) in freshly distilled pyridine (30 mL), dichlorophenylphosphine (0.773 mL, 5.70 mmol) was added slowly under argon, and the reaction was kept under stirring at 70 °C. After 1 h S_8_ (269 mg, 1.05 mmol) was added and the reaction was stirred for another hour at 50 °C. After cooling to room temperature, the solution was treated with an excess of H_2_O_2_, added dropwise at 0 °C, and the reaction was stirred for 1 h at room temperature. H_2_O was then added to the reaction, favoring the formation of a white precipitate that was filtrated and washed with water. The crude was purified by column chromatography (SiO_2_, CH_2_Cl_2_) to give the desired product with a 60% yield. ^1^H-NMR (CDCl_3_, 300 MHz): δ (ppm) 8.26–8.19 (m, 8H, PSArH*_o_*), 7.63–7.55 (m, 12H, PSArH*_m_* + PSArH*_p_*), 7.28 (s, 4H, ArH*_down_*), 7.76 (bt, 4H, ArC*H*), 2.35–2.30 (m, 8H, C*H_2_*CH_2_CH_3_), 2.10 (s, 12H, ArC*H_3_*), 1.43–1.41 (m, 8H, CH_2_C*H_2_*CH_3_), 1.11–1.06 (m, 12H, CH_2_CH_2_C*H_3_*). ^31^P-NMR (CDCl_3_, 162 MHz): δ (ppm) 77.79 (s, 4P, P=S). ESI-MS: *m*/*z* 1287.8 [M+Na]^+^.

#### 3.2.5. Cavitand **3POiii1CH_2_[C_3_H_7_**, **CH_3_**, **Ph]**

In a Schlenk tube, to a solution of a tri-bridged resorcinarene **Tiii[C_3_H_7_**, **CH_3_**, **Ph]** (200 mg, 0.185 mmol) in dry DMA (15 mL), K_2_CO_3_ (153 mg, 1.11 mmol) and CH_2_BrCl (38 μL, 0.58 mmol) were added under argon and the reaction was kept under stirring at 90 °C for 3 h. After removal of the solvent *in vacuo*, the crude was extracted with CH_2_Cl_2_/acidic H_2_O (HCl 1M) and dried at the vacuum pump, obtaining the product in quantitative yield without further purification. ^1^H-NMR (CDCl_3_, 300 MHz): δ (ppm) 8.03 (m, 6H, P(O)ArH*_o_*), 7.66 (m, 3H, P(O)ArH*_p_*), 7.57 (m, 6H, P(S)ArH*_m_*), 7.29 (s, 2H, ArH*_down_*), 7.26 (s, 2H, ArH*_down_*), 5.69 (d, 1H, OC*H_2(out)_*O, *J* = 7.4 Hz), 5.03 (d, 1H, OC*H_2(in)_*O, *J* = 7.4 Hz), 4.71 (m, 4H, ArC*H*), 2.16–2.13 (s+s, 12H, ArC*H_3_*) 1.41–1.38 (m, 8H, CH_2_C*H_2_*CH_3_), 1.12–1.08 (m, 12H, CH_2_CH_2_C*H_3_*). ^31^P-NMR: (CDCl_3_, 162 MHz): δ (ppm) 7.40 (s, 1P); 6.73 (s, 2P). ESI-MS (*m*/*z*): 1114 [M+Na]^+^.

### 3.3. Crystal Structure of **1POi3PSiii[C_2_H_5_**, **H**, **Ph]**

The crystal structure of compound **3PSiii1POi[C_2_H_5_**, **H**, **Ph]·3C_4_H_8_O** was determined by X-ray diffraction methods. Crystal data and experimental details for data collection and structure refinement are reported in Table 2.

**Table 2:** Crystal data and structure refinement information for **1POi3PSiii[C_2_H_5_**, **H**, **Ph]·3C_4_H_8_O**.

Empirical Formula	C_72_H_76_O_12_P_4_S_3_
Formula weight (g·mol^−1^)	1353.39
Temperature (K)	190(2)
Crystal system	Triclinic
Space group	*P*–1
*a* (Å)	11.961(2)
*b* (Å)	16.442(2)
*c* (Å)	19.828(3)
α (°)	109.717(3)
β (°)	101.308(3)
γ (°)	95.779(3)
*V* (Å^3^)	3540.3(9)
*Z*	2
ρ_calcd_	1.270
μ (mm^−1^)	0.254
*F*(000)	1424
*θ* for data collection (°)	1.13–26.55
Reflections collected/unique	14587/14587 [R(int) = 0.0]
Observed reflections [F_o_ > 4σ(F_o_)]	8806
Completeness to theta	98.8
Data/restraints/parameters	14587/0/720
Goodness-of-fit on *F*^2 a^	1.056
Final *R* indices [F_o_ > 4σ(F_o_)] ^b^	R1 = 0.0658, wR2 = 0.1991
*R* indices (all data)	R1 = 0.1000, wR2 = 0.2193
largest diff. peak and hole (*e* Å^3^)	1.027 and −0.568

^a^ Goodness-of-fit S = [Σw(F_o_^2^ − F_c_^2^)^2^/(n − p)]1/2, where n is the number of reflections and p the number of parameters. ^b^ *R*_1_ = Σ║F_o_│ − │F_c_║/Σ│F_o_│, *wR*_2_ = [Σ[w(F_o_^2^ − F_c_^2^)^2^]/Σ[w(F_o_^2^)^2^]]^1/2^.

Intensity data and cell parameters were recorded at 190(2) K on a Bruker Smart AXS 1000 (MoKα radiation λ = 0.71073 Å) equipped with a CCD area detector and a graphite monochromator. The raw frame data were processed using SAINT and TWINABS to yield the reflection data files [13,14,15]. The structure was solved by Direct Methods using the SIR97 program [16] and refined on F_o_^2^ by full-matrix least-squares procedures, using the SHELXL-97 program [17] in the WinGX suite v.1.80.05 [18].

In view of the presence of disordered THF molecules which could not be properly modelled, the structure was subjected to the program SQUEEZE [19]. The program calculated a void volume of 732.3 Å^3^ and 158 electrons per unit cell, which corresponds to four THF molecules per unit cell. All non-hydrogen atoms were refined with anisotropic atomic displacements except in the case of one disordered phenyl group at the upper rim. The hydrogen atoms were included in the refinement at idealized geometry (C-H 0.95 Å) and refined “riding” on the corresponding parent atoms. The weighting schemes used in the last cycle of refinement were *w* = 1/[σ^2^*F_o_*^2^ + (0.1346*P*)^2^], where *P =* (*F_o_*^2^ + 2*F_c_*^2^)/3.

Crystallographic data (excluding structure factors) for the structure reported have been deposited with the Cambridge Crystallographic Data Centre as supplementary publication no. CCDC-1048659 and can be obtained free of charge on application to the CCDC, 12 Union Road, Cambridge, CB2 IEZ, UK (fax: +44-1223-336-033; e-mail deposit@ccdc.cam.ac.uk or http://www.ccdc.cam.ac.uk).

## 4. Conclusions

The present study sheds light on the complexation behavior of mixed-bridged P=O/P=S cavitands toward *N,N*-methyl alkyl ammonium salts. The progressive substitution of P=O bridges with P=S ones allows to single out their respective influence on the complexation behavior. The P=S bridge, despite being unable of H-bonding and inefficient in cation-dipole interactions, plays a non-innocent role in the complexation of **1**. The replacement of one/two vicinal P=O with P=S does not reduce the Kass values, provided that the two synergistic H-bonds with the residual P=O are maintained (AB substitution pattern). A single replacement is even slightly beneficial, as shown by the **1@3POiii1PSi** case. The bulkiness of P=S bridges helps in narrowing the cavity mouth, affecting desolvation and guest mobility.

The overall picture emerging from this study gives a privileged position to the dual H-bonding mode available for secondary ammonium ions in complexation with the P=O bridges. The role of CH_3_-π interactions has not been underlined here for two reasons: they are present in all five complexation efficient cavitands and their role is diminished by the organic solvent (DCE in this case). However CH_3_-π interactions are pivotal for selectivity once the cavitands are operating in water or at the water-solid interface [4,5], since, unlike H-bonds, they are unaffected by the presence of water.

## Supplementary Materials

Supplementary materials can be accessed at: http://www.mdpi.com/1420-3049/20/03/4460/s1.
