# Supplementary material for: The Effect of Number and Position of P=O/P=S Bridging Units on Cavitand Selectivity toward Methyl Ammonium Salts
Source: Molecules. 2015 Mar 10;20(3):4460–72. doi: 10.3390/molecules20034460 (PMC6272763; doi:10.3390/molecules20034460)
Supplement: Supplementary file 1 [file molecules-20-04460-s001.pdf]

Supplementary Materials

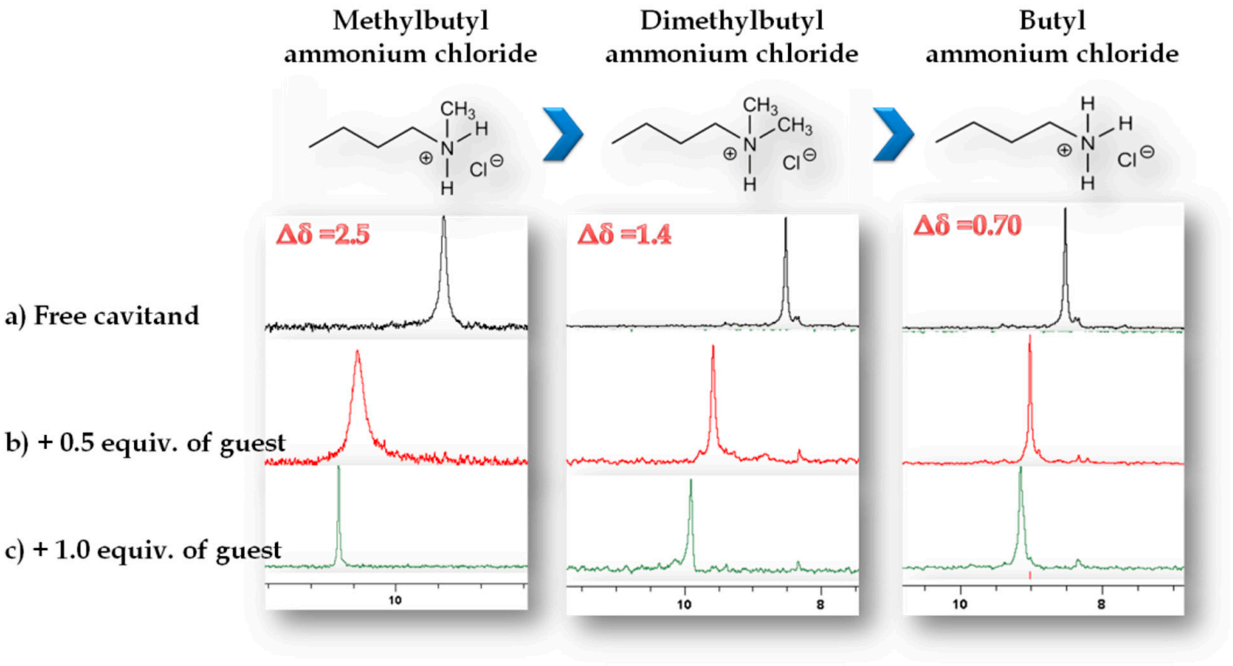

Figure S1. ^{31}P NMR of **Tiiii[C_3H_7, CH_3, Ph]·methylbutyl/dimethylbutyl/butyl ammonium chloride** qualitative complexation test: (**a**) spectra of the free cavitand; (**b**) spectra recorded after the addition of 0.5 equivalents of the guest; (**c**) spectra recorded after the addition of 1.0 equivalent of the guest. All spectra were recorded in MeOD.

ITC curves and fittings

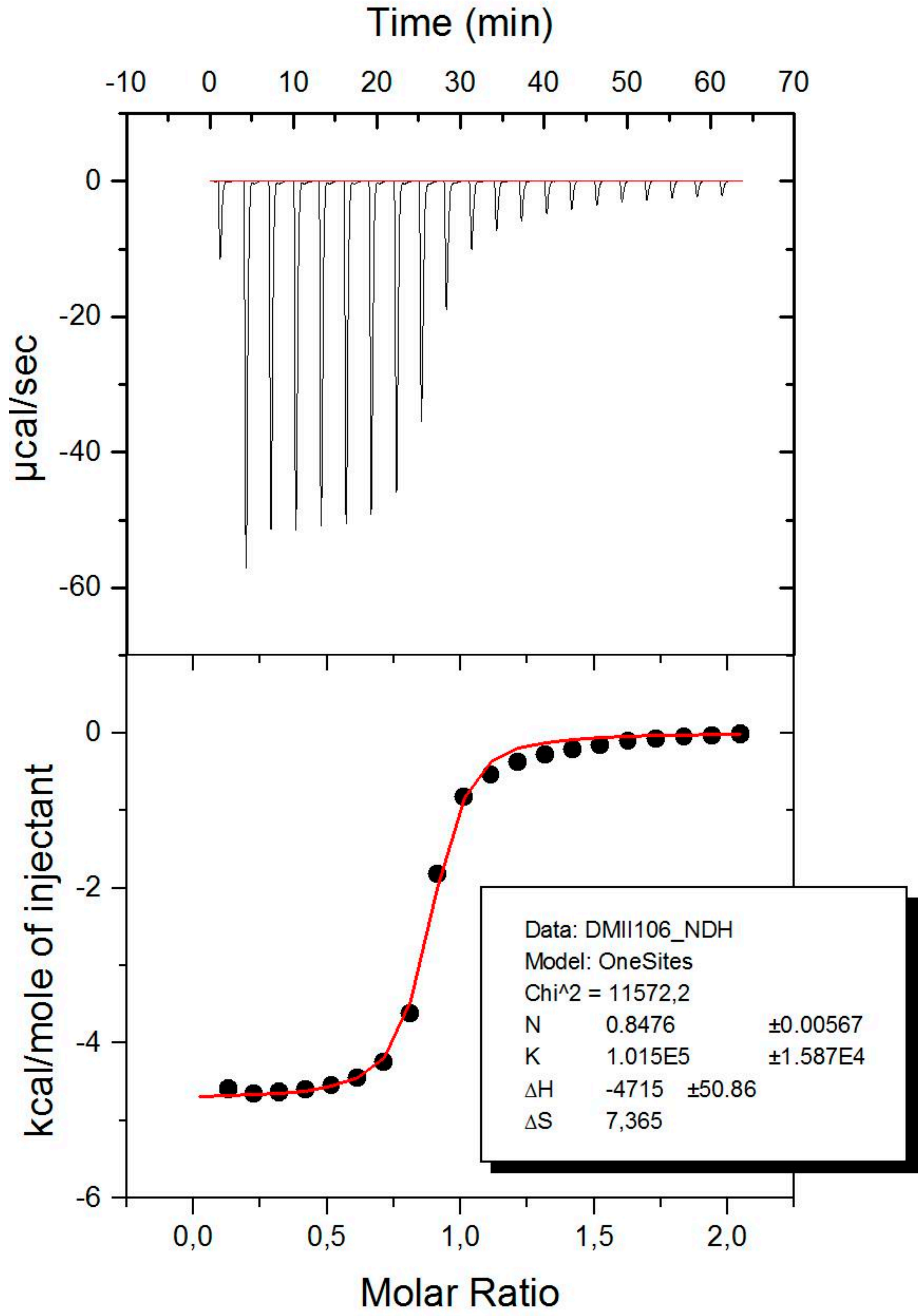

Figure S2. ITC trace and fitting of **1@Tiiii**.

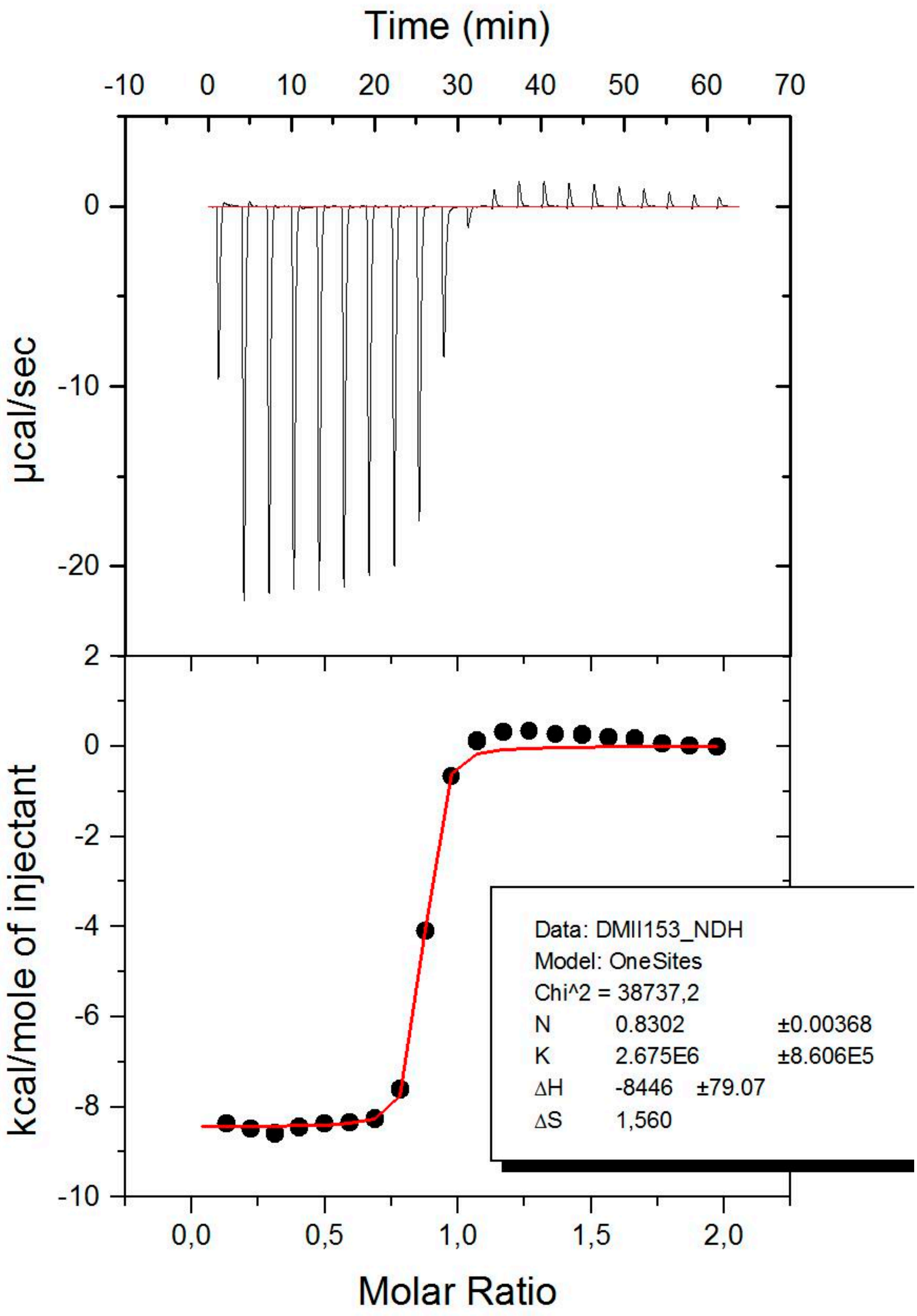

Figure S3. ITC trace and fitting of **1@3POiii1PSi**.

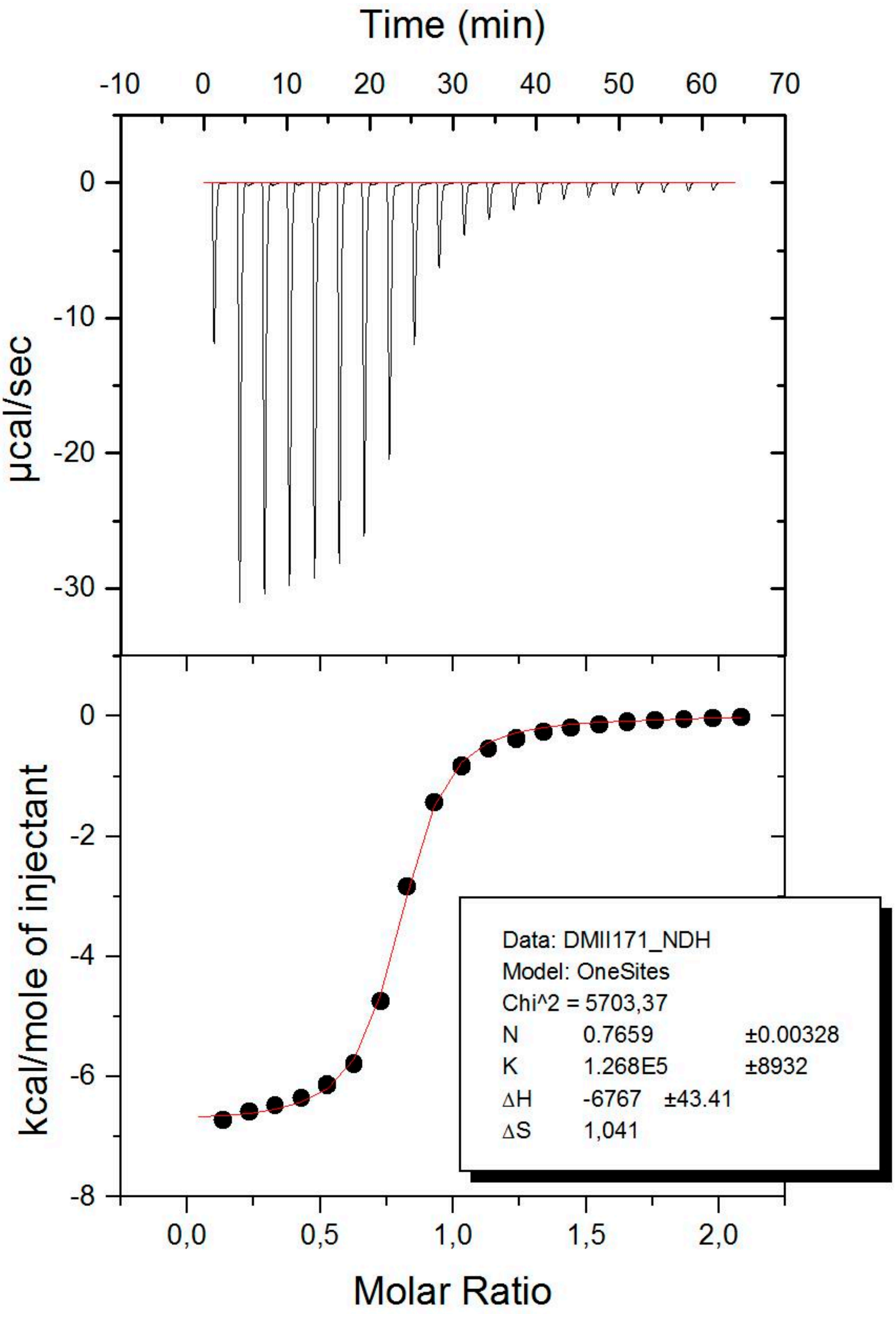

Figure S4. ITC trace and fitting of **1@AB2POii2PSii** first run.

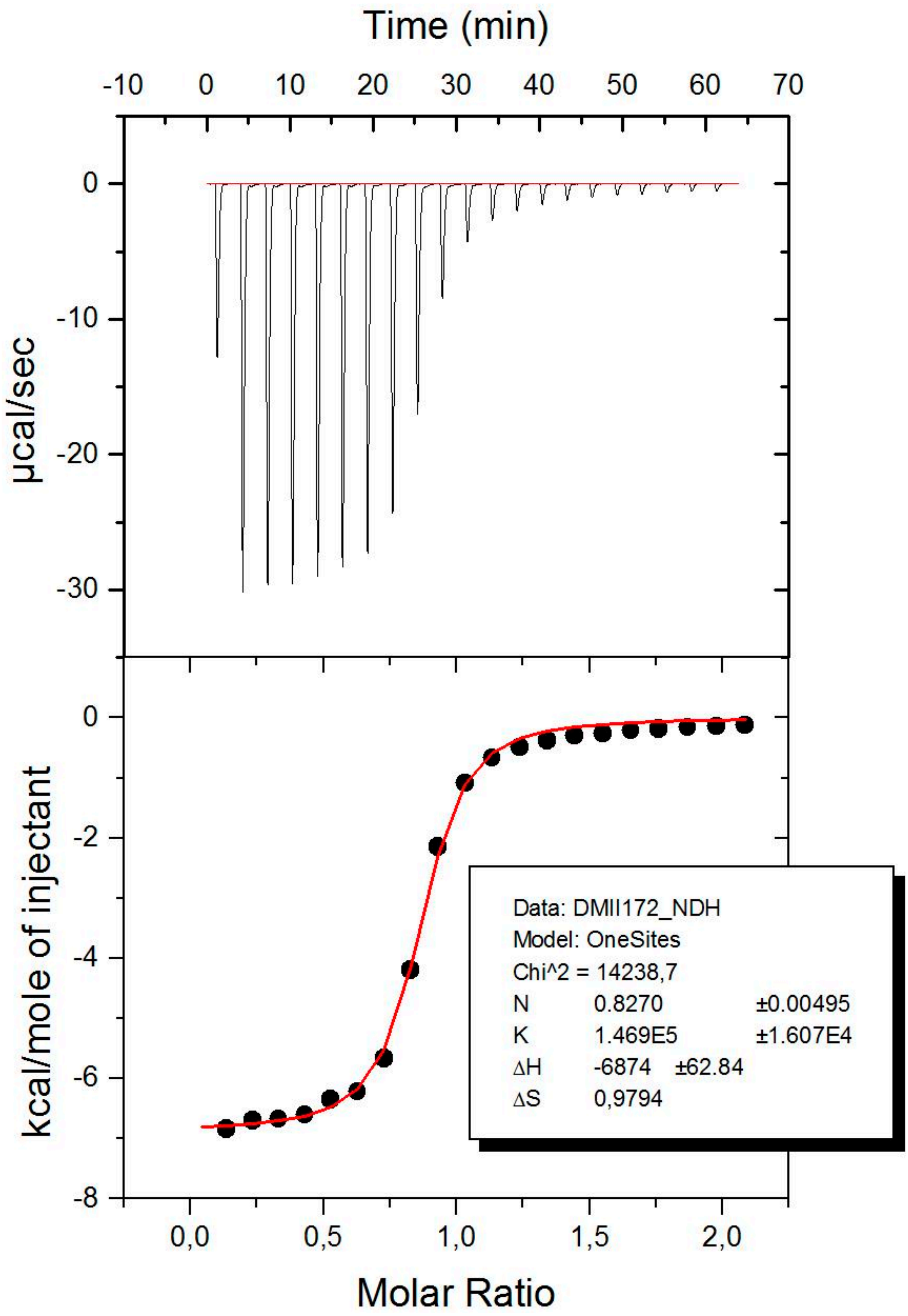

Figure S5. ITC trace and fitting of **1@AB2POii2PSii** second run.

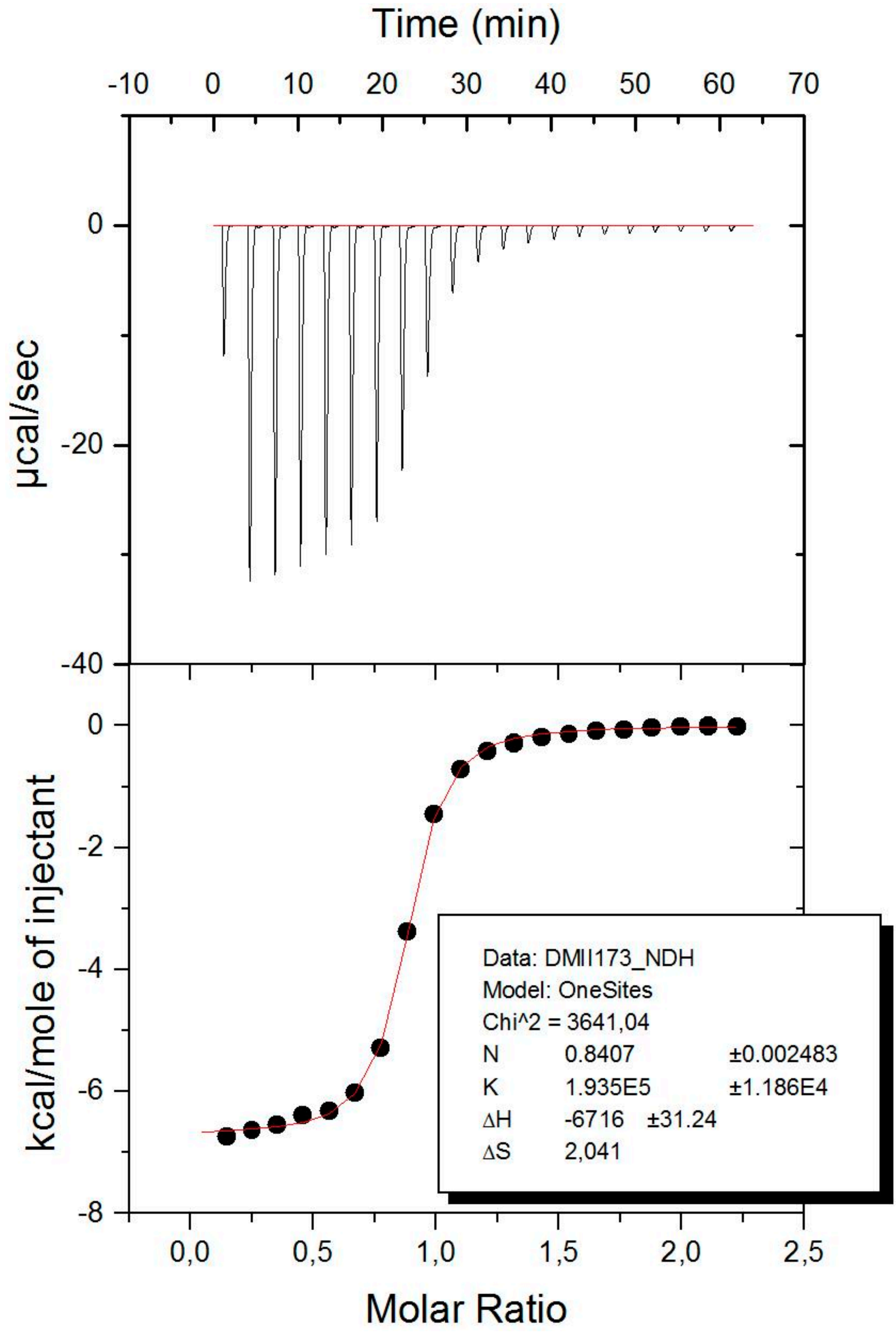

Figure S6. ITC trace and fitting of **1@AB2POii2PSii** third run.

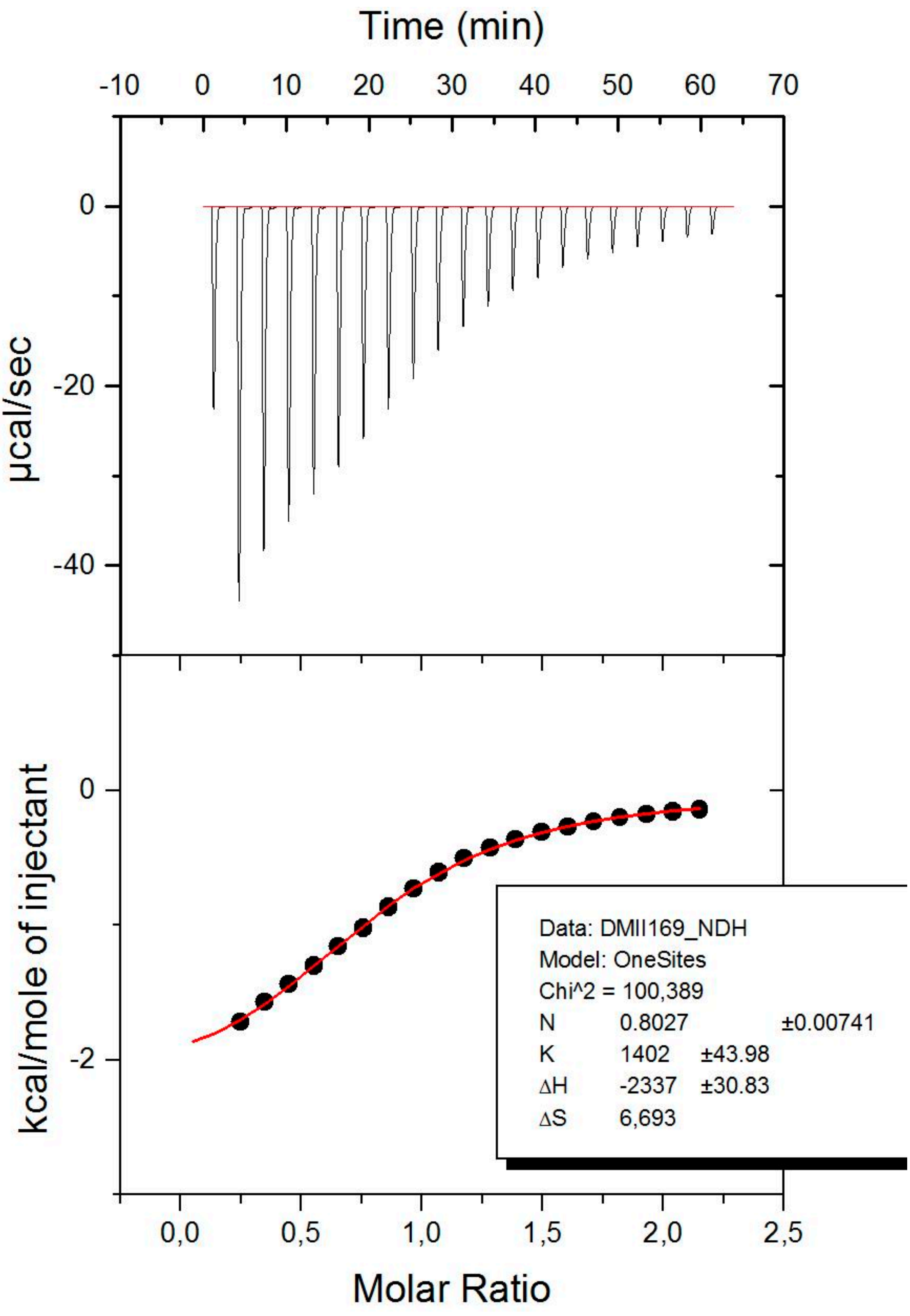

Figure S7. ITC trace and fitting of **1@AC2POii2PSii**.

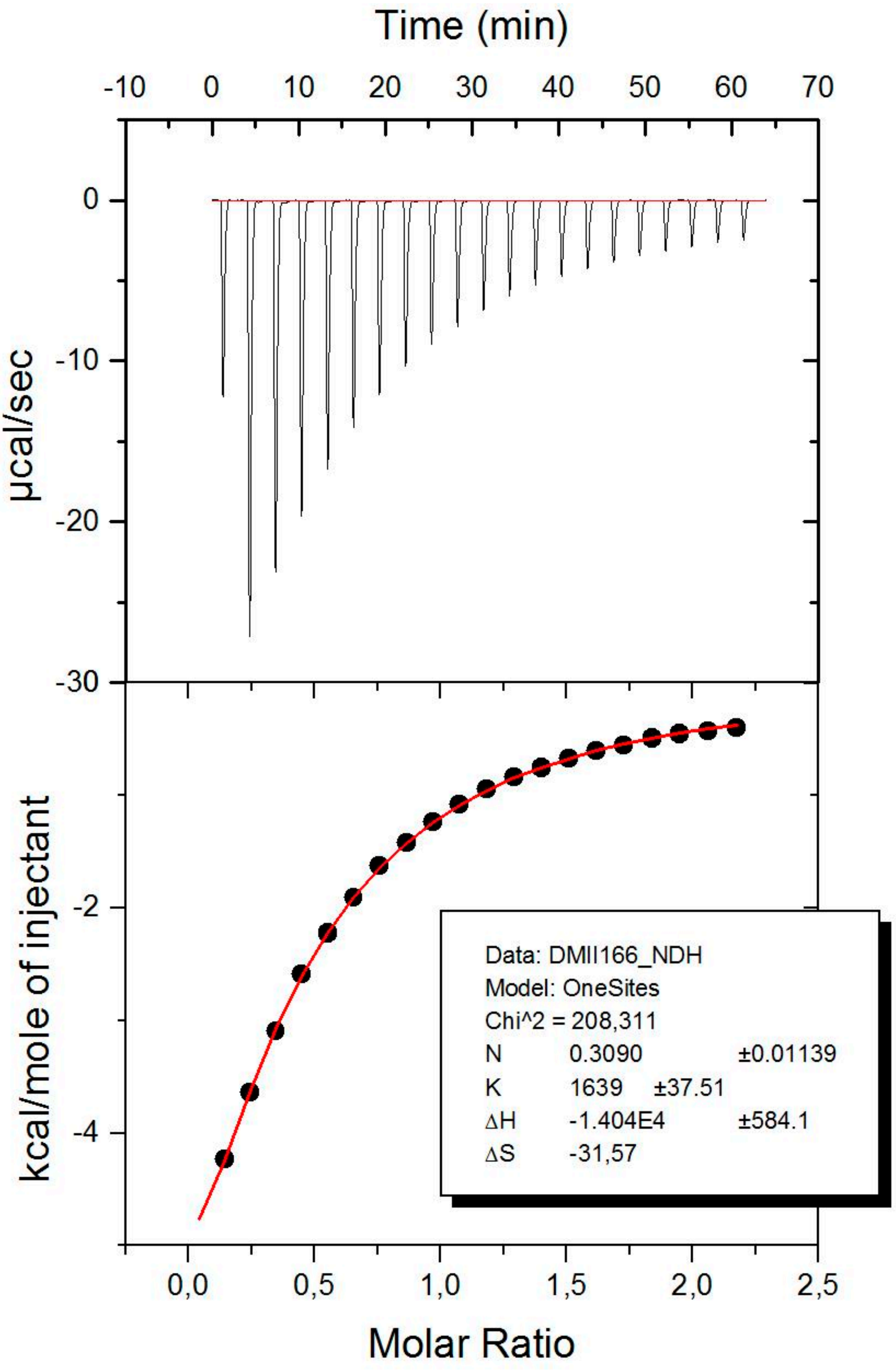

Figure S8. ITC trace and fitting of **1@1POi3PSiii**.

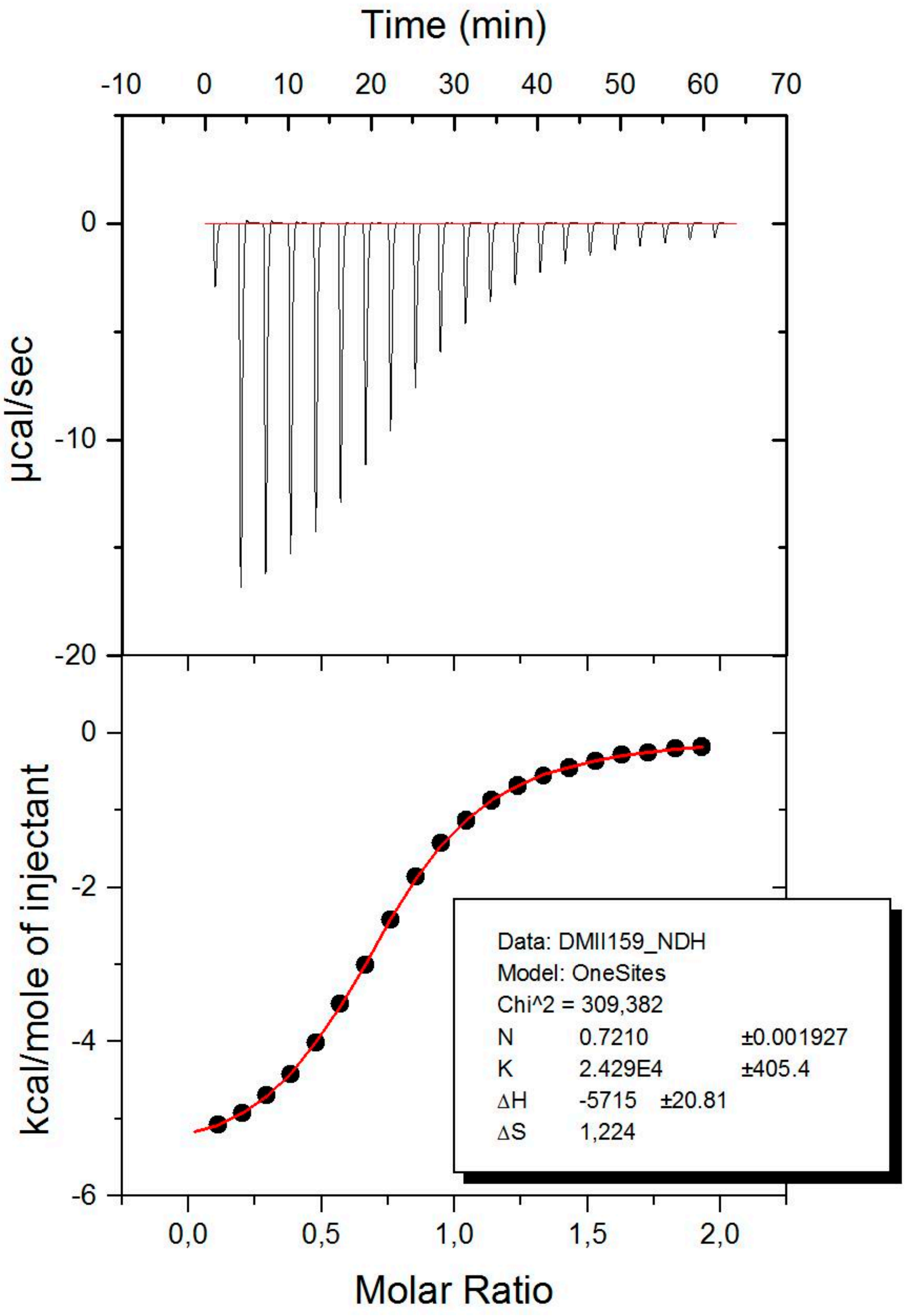

Figure S9. ITC trace and fitting of **1@3POiii1CH$_2$**.

^{1}H and ^{31}P-NMR of the new cavitands

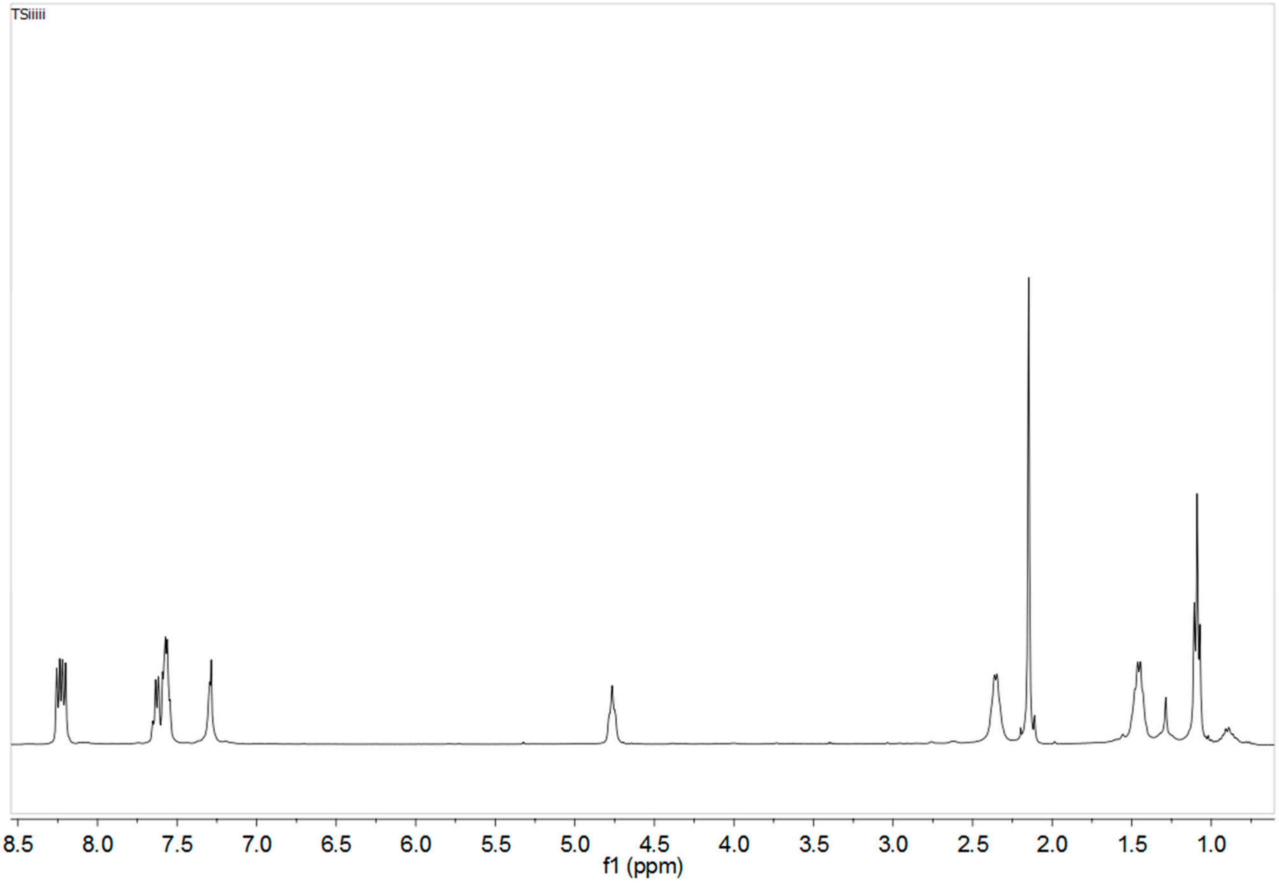

Figure S10. ^{1}H-NMR of TSiiii.

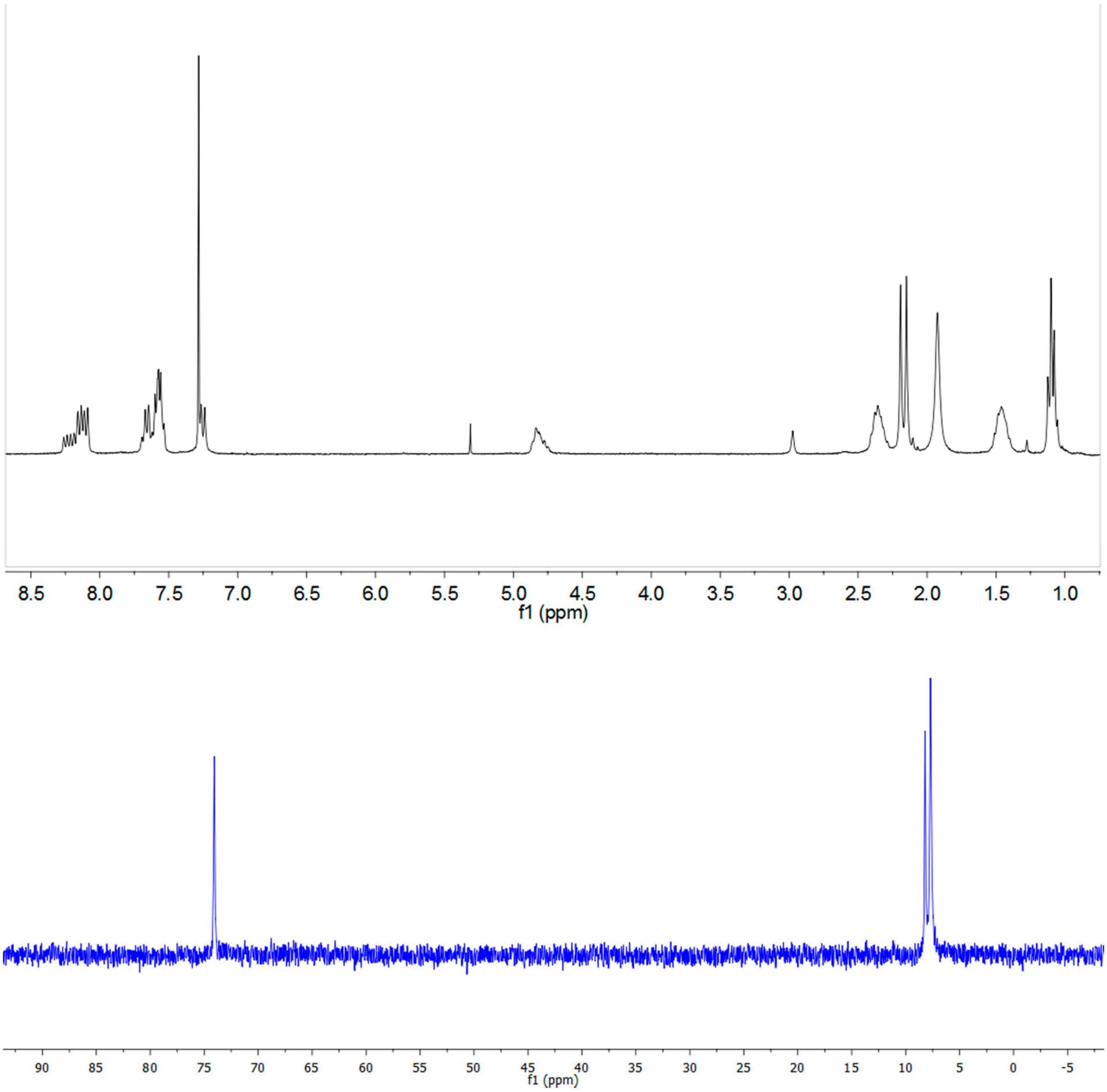

Figure S11. ^{1}H and ^{31}P-NMR of **3POiii1Psi**.

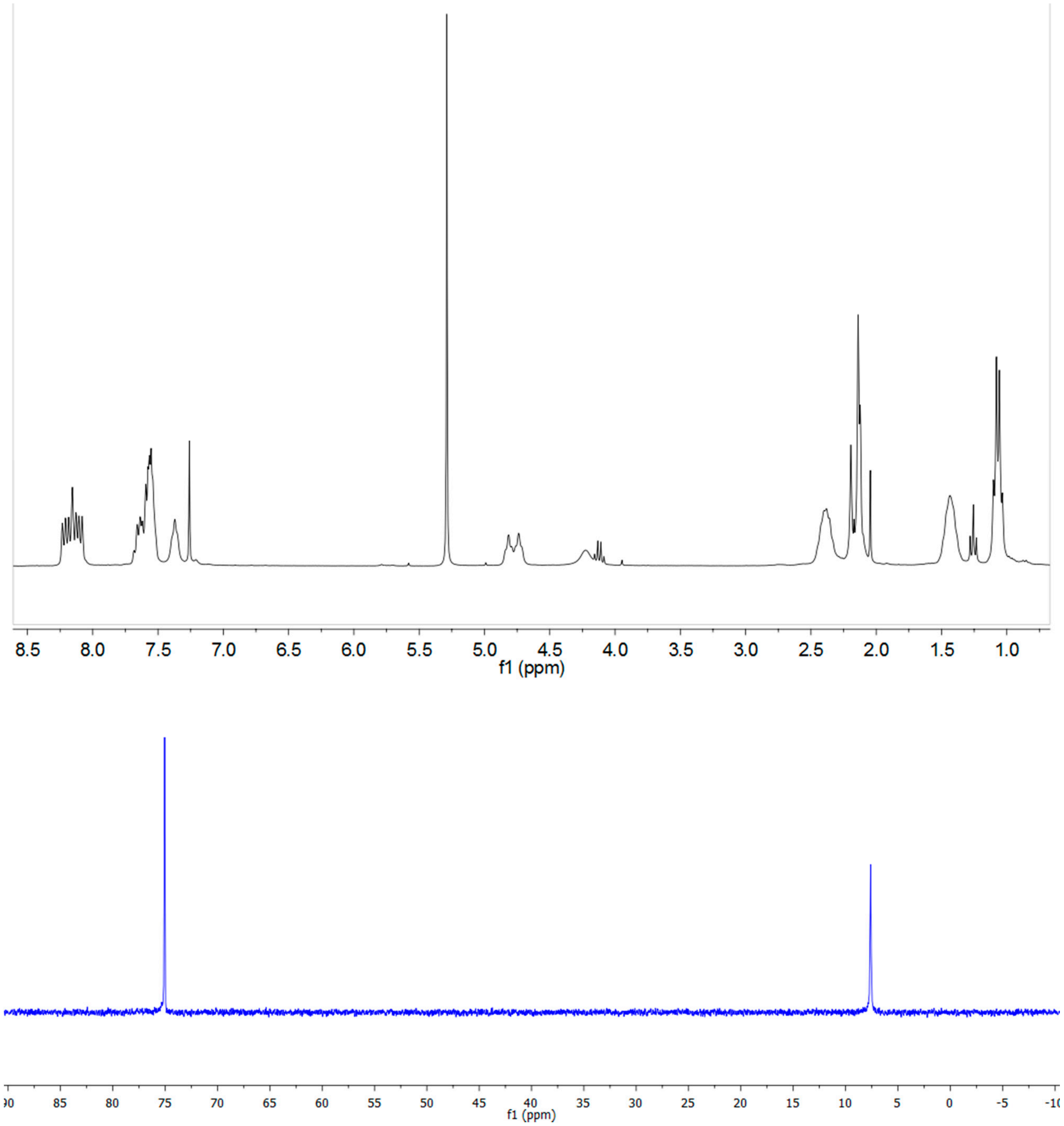

Figure S12. ^{1}H and ^{31}P-NMR of AB2POii2PSii.

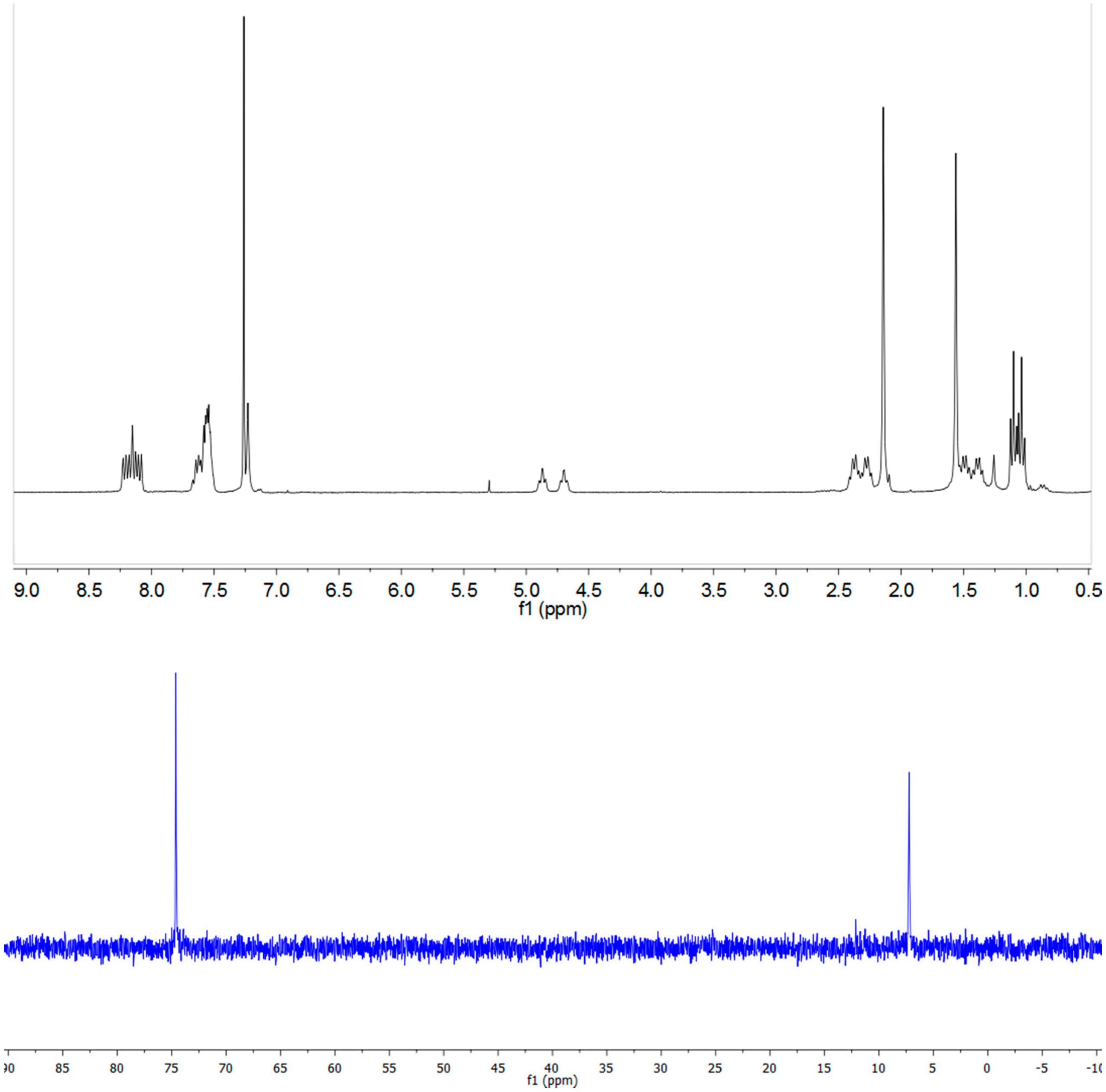

Figure S13. ^{1}H and ^{31}P-NMR of AC2POii2PSii.

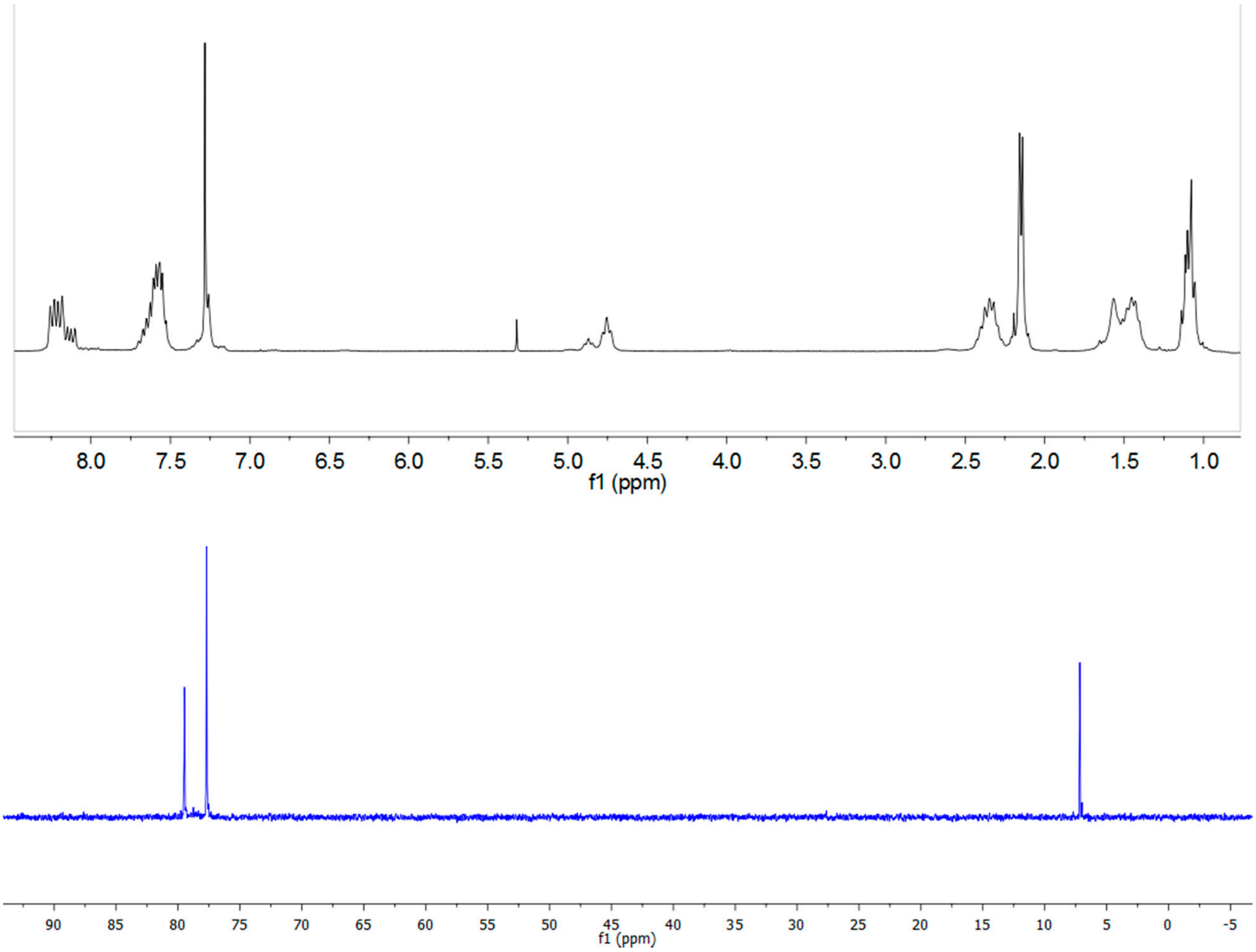

Figure S14. ^{1}H and ^{31}P-NMR of **1POi3PSiii**.

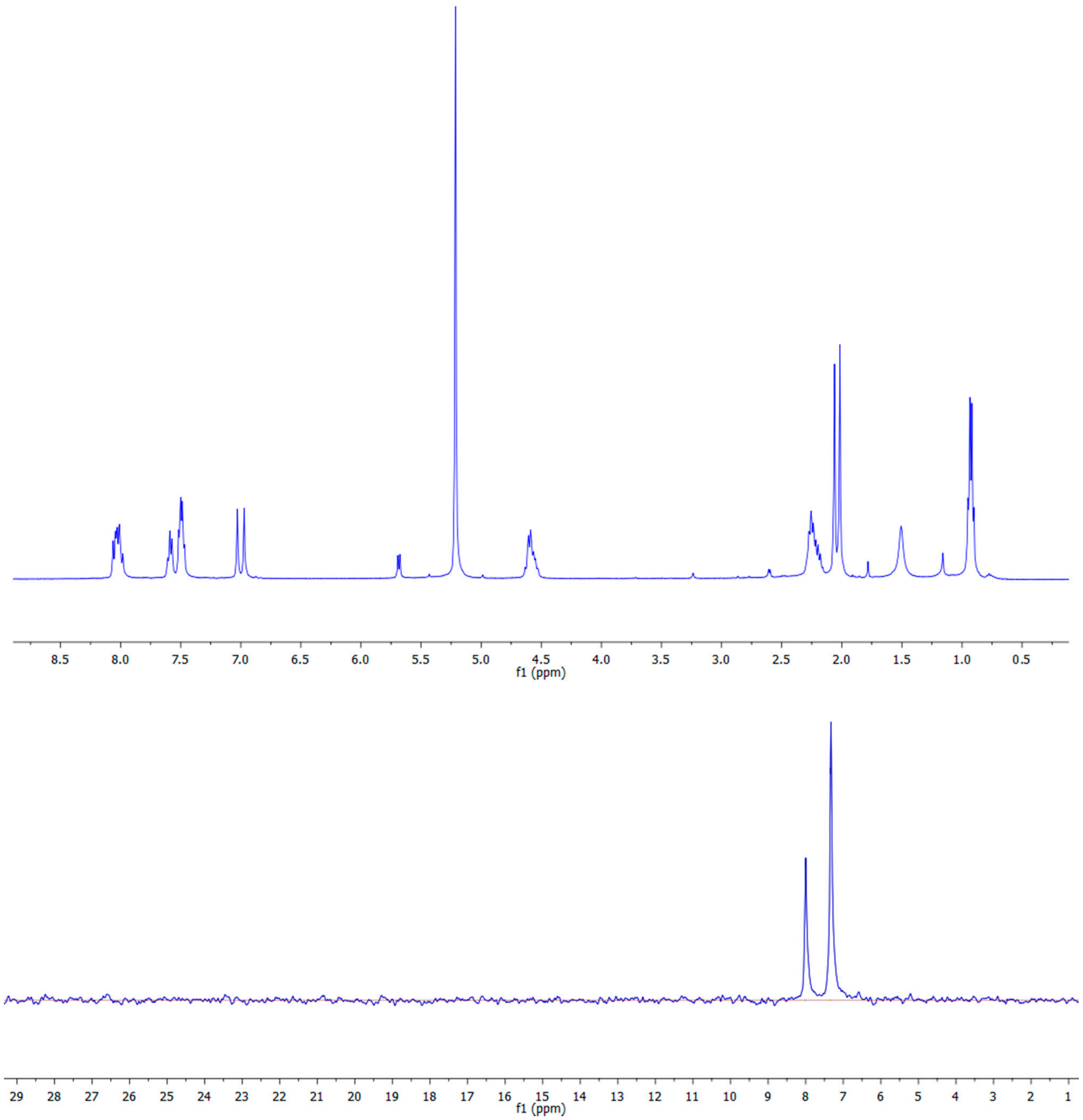

Figure S15. ^{1}H and ^{31}P-NMR of **3POiii1CH$_2$**.
